# Revisional Single-Anastomosis Gastric Bypass for a Patient With Severe Protein-Calorie Malnutrition: A Case Report

**DOI:** 10.7759/cureus.34987

**Published:** 2023-02-14

**Authors:** José S Verboonen Sotelo, Jeffry Romero Manzano, Guillermo Vega Tostado, Jose Aldo Guzmán Barba, Isaac Esparza Estrada

**Affiliations:** 1 Bariatric Surgery, Obesity Goodbye Center, Tijuana, MEX

**Keywords:** reversal surgery, revision surgery, protein-caloric malnutrition, bariatric surgery, mini gastric bypass, obesity

## Abstract

Obesity is a chronic disorder with numerous manifestations as well as pandemic occurrence on a global scale. One-anastomosis gastric bypass (OAGB) is a safe and effective surgical procedure for the treatment of obesity and metabolic diseases. Revision surgery may be necessary due to postoperative issues, such as protein-calorie deficiency, weight increase, or inadequate weight loss. This case describes a 40-year-old female patient who came to our service due to protein-caloric malnutrition, with a history of OAGB. The patient underwent revision surgery for OAGB reversal. One year after surgery the patient had a body mass index (BMI) of 25; today she is healthy, consumes a regular diet, and has no associated complications.

## Introduction

Obesity is a chronic disorder with numerous manifestations and is considered to be a global pandemic [1]. Over the past few decades, the discipline of bariatric surgery has grown significantly owing to its effectiveness in treating specific disorders, such as obesity, diabetes mellitus, and hypertension [2]. According to the World Health Organization (WHO), being overweight or obese results in at least 2.8 million fatalities per year. Globally, obesity prevalence increased, with 13% of the population being obese [3]. There is greater morbidity and mortality in patients with severe obesity, especially classes II (BMI 35.0 to 39.9) and III (BMI greater than 40). In addition, obesity is a recognized risk factor for the development of comorbid conditions such as cardiovascular disease, type 2 diabetes mellitus, malignancy, asthma, osteoarthritis, chronic back pain, obstructive sleep apnea, non-alcoholic fatty liver disease, and gallbladder diseases [4].

One-anastomosis gastric bypass (OAGB), also known as mini-gastric bypass (MGB), is a safe and effective surgical procedure for treating obesity and metabolic diseases [5]. Due to increased initial bariatric surgeries, surgeons are now treating more patients who have already undergone failed and/or complicated bariatric procedures. Revision surgery may be necessary due to postoperative issues, such as protein-calorie shortage, weight increase, or inadequate weight loss [6].

## Case presentation

A 40-year-old female patient, native to and resident of Mexico, had no allergies or chronic degenerative diseases and no history of alcohol consumption or smoking. Surgical history included a gastric band at 30 years of age, gastric sleeve at 36 years of age, and OAGB at 39 years of age. Her height was 1.62 m, weight 67 kg, and had a BMI of 25.5. The patient arrived at our hospital with poor general condition, oral intolerance, asthenia, and adynamia. On physical examination, the patient was oriented, uncooperative, and had a negative attitude. Cardiopulmonary and abdominal examination without pathological data showed lower extremities with edema covering two-thirds. Relevant laboratory studies: hemoglobin 8.6 g/dl, hematocrit 29%, leukocytes 4,700 mm3, platelets 179,000 mcL, albumin 2.1 g/dl, and total proteins 4.6 g/dl. Protein-calorie malnutrition was diagnosed. She did not tolerate the oral route, so it was decided to start parenteral nutrition by central venous catheter, smofkabiven at 82 ml per minute by continuous infusion pump; additionally, vitamin C was added and a globular package was transfused. After 10 days, the patient was stabilized and revision surgery for OAGB reversal was scheduled. Relevant laboratory studies: hemoglobin 9.6 g/dl, hematocrit 32%, leukocytes 5,500 mm3, platelets 212,000 mcL, and albumin 2.5 g/dl.

Surgical report

**Figure 1:**
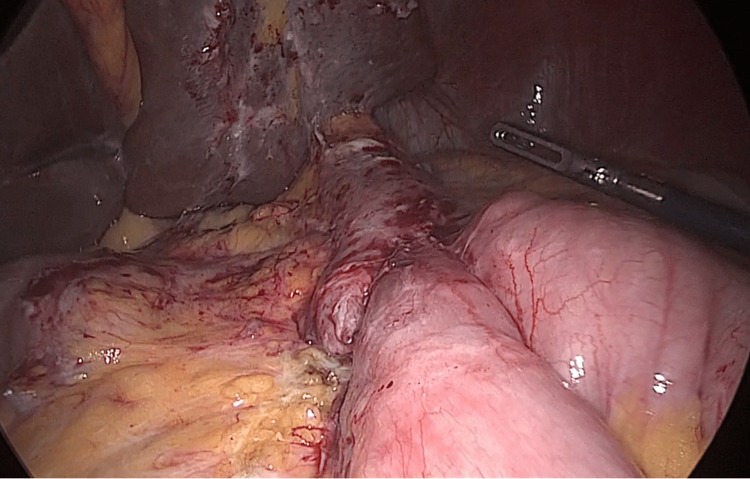
Pouch and gastrojejunal anastomosis We identified the pouch and gastrojejunal anastomosis from the previous mini-bypass surgery

**Figure 2:**
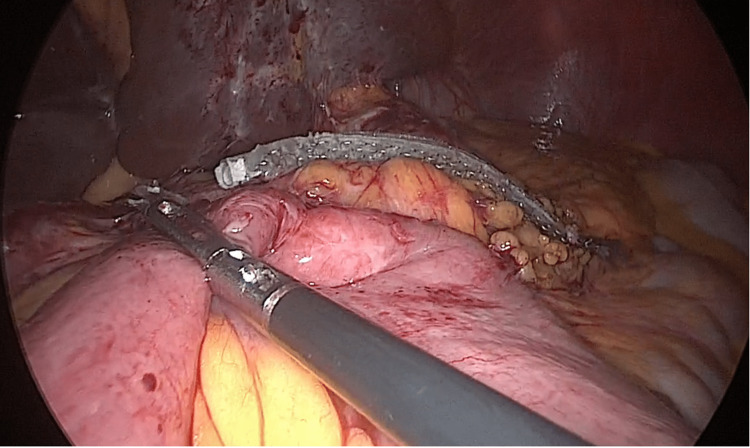
Gastrojejunal anastomosis Anastomosis from the mini-gastric bypass is clamped with a reinforced cartridge

**Figure 3:**
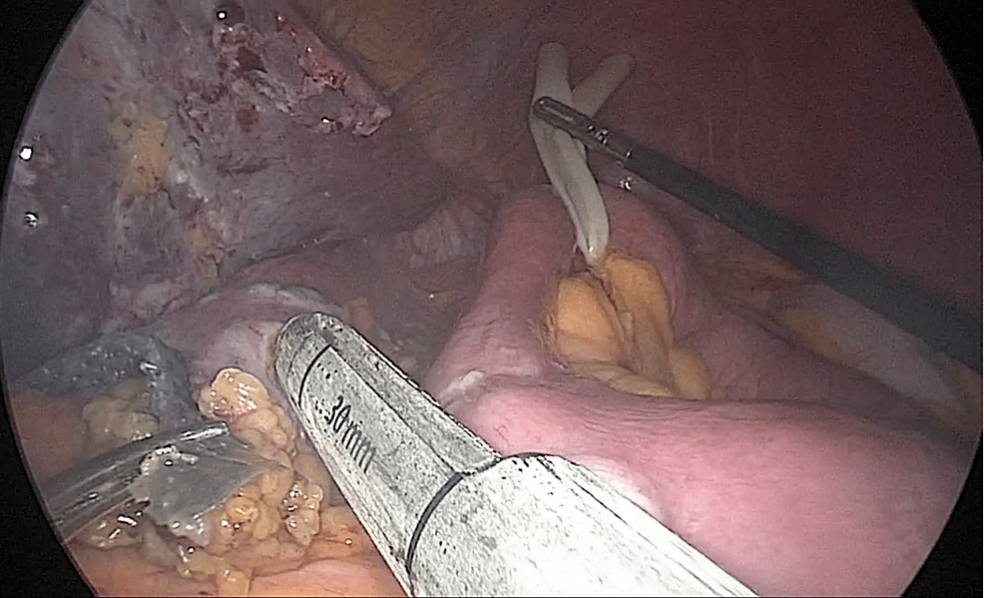
New anastomosis We counted the intestine in order to increase the absorptive portion, then we performed the new anastomosis

**Figure 4:**
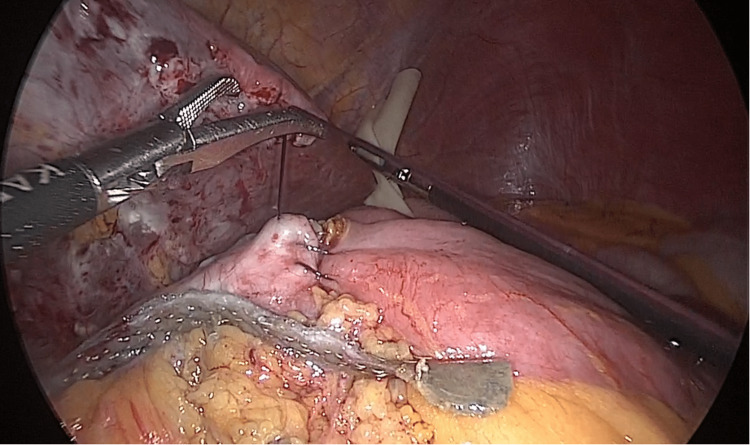
New anastomosis closure Finally we closed the top of the new anastomosis with Monocryl 2-0

The patient was placed in a supine position under general anesthesia, asepsis and antisepsis were performed, laparoscopic equipment was connected, and the pouch was localized. We counted from the Treitz segment that was anastomosed to the stomach (290 cm) (Figures 1-2), then counted the rest of the small intestine which measured 320 cm, for a total length of 610 cm. Next, we placed a black 60 mm reinforced staple between the pouch and the small intestine, where the previous anastomosis was, and separated the pouch from the first 290 cm anastomosis. We counted back 130 cm to perform the new anastomosis in order to increase the absorptive portion. The anastomosis was performed with a COVIDIEN purple staple of 30 mm (Figure 3), then closed manually by laparoscopy with Prolene 2-0 continuous points (Figure 4). Hermeticity tests were performed with methylene blue and air; both were negative so the cavity was aspirated and a closed drain (Jackson Pratt) was placed at the site of anastomosis. The operative time was 82 minutes and the blood loss was 40 mL.

The patient had a normal postoperative course with no complications, she stayed seven days in the hospital before her discharge. One year after surgery the patient had a BMI of 25; today she is healthy, consumes a regular diet, and has no complications. Laboratory studies one year after surgery: hemoglobin 12.6 g/dl, hematocrit 41.2%, leukocytes 8,500 mm3, platelets 280,000 mcL, albumin 4.2 g/dl, and total proteins 6.7 g/dl.

## Discussion

Obese patients frequently experience nutritional inadequacy before and after bariatric surgery. Therefore, it is crucial to provide patients with continuous metabolic and nutritional monitoring, especially during surgeries that result in malabsorptive outcomes [6]. After OAGB, malnutrition is not unusual (0.2-10.8%) [7].

OAGB is similar to roux-en-Y gastric bypass (RYGB) in terms of the percentage of excess BMI lost after two years. This result is consistent with the 64.4% excess weight reduction with OAGB reported by Lee et al. in the first randomized trial comparing these two procedures, published in 2005 [8]. Ruiz-Tovar et al. compared OAGB with RYGB and sleeve gastrectomy and discovered that two years after the procedure, the OAGB group lost a much larger proportion of extra body mass than the RYGB group [9].

Recent studies also detailed serious nutritional problems following OAGB. A report of 26 patients with severe and refractory malnutrition following OAGB showed normal anatomy reversal. Genser et al. found that the mean time between OAGB and reversal surgery was 20.9 months [10]. Furthermore, Poghosyan et al. included 17 patients who underwent OAGBs modified to RYGBs due to serious problems; 10 (59%) patients received preoperative nutritional assistance for undernutrition [11]. A meta-analysis by Magouliotis et al. also found a higher prevalence of malnutrition after OAGB than after RYGB [12].

After obesity and metabolic surgery, it is crucial to identify, treat, and manage any unique long-term consequences to maintain a patient’s quality of life. The prevalence of revisional bariatric surgery (RBS) has increased over time, with a prevalence of 13.6% in the United States of America in 2016. OAGB/MGB is one of the most popular bariatric procedures and has a low RBS incidence [13,14]. The major cause of reversal surgery is malnutrition with hypoalbuminemia and associated clinical symptoms. For this reason, protein-calorie deficiency in all OAGB/MGB patients should receive extra attention during follow-up to detect postoperative severe malnutrition [15].

## Conclusions

A major cause of revision surgery is protein-calorie malnutrition with hypoalbuminemia and associated clinical symptoms. To diagnose postoperative severe malnutrition early, extra attention should be devoted to all OAGB/MGB patients during their hospital stay and follow-up. Revision surgery has proven to be an efficient and safe procedure with good outcomes; thus, it must be considered when complications in bariatric patients appear and other less invasive treatment options fail. However, due to its difficulty, it should only be performed by experienced surgeons.
